# Hemophagocytic Lymphohistiocytosis Secondary to Epstein-Barr Virus: A Case Report

**DOI:** 10.7759/cureus.89008

**Published:** 2025-07-29

**Authors:** Laura D Lopez, Emily Gioe, Rabar Mudhher, Veron Browne, Hosein Shokouh-Amiri, Gazi B Zibari

**Affiliations:** 1 Internal Medicine, Surgery, Universidad Nacional Autonoma de Honduras, San Pedro Sula, HND; 2 Medicine, Edward Via College of Osteopathic Medicine, Monroe, USA; 3 Research, Willis Knighton Health, Shreveport, USA; 4 Transplant Hepatology, Willis Knighton Health, Shreveport, USA; 5 Transplant Surgery, Willis Knighton Health, Shreveport, USA

**Keywords:** cytotoxic, epstein-barr virus, hemophagocytic lymphohistiocytosis, hlh-2004 protocol, lymphadenopathy

## Abstract

Hemophagocytic lymphohistiocytosis (HLH) is a rare disease more commonly found in neonates and adolescents. It can be classified as primary/genetic or acquired following viral infection, lymphomas, or autoimmune diseases. There is not enough evidence of the prevalence of this disease in adults, but, when diagnosed, it is most commonly secondary to viruses, such as Epstein-Barr virus (EBV). We report an adult case of HLH secondary to EBV in a 27-year-old Hispanic male with no significant past medical history, who presented to the emergency department with constant fatigue, myalgia, nausea, vomiting, right upper quadrant abdominal pain, and dizziness. He met six out of eight potential criteria for HLH, including splenomegaly, fever, cytopenia, hypofibrinogenemia, hyperferritinemia, and positive sCD25. He was diagnosed based on the HLH-2004 diagnostic criteria and was treated with the HLH-94 protocol. After being hospitalized for almost two months, the patient failed to respond to treatment and passed away. Prompt treatment of HLH is advised due to rapid deterioration.

## Introduction

Hemophagocytic lymphohistiocytosis (HLH) is an exaggerated and potentially life-threatening inflammatory syndrome characterized by an uncontrolled and excessive activation of abnormal macrophages and natural killer T-cells. These cells release many cytokines, leading to tissue damage and multiple organ failure [1]. The term "hemophagocytosis" describes the pathognomonic findings where highly activated macrophages take up different cells, including lymphocytes, erythrocytes, leukocytes, and platelets in different tissues, producing excessive cytokines and an uncontrolled inflammatory reaction. This disease can be classified into two categories: primary or familial lymphohistiocytosis and secondary lymphohistiocytosis [1,2]. Primary HLH is due to a defect in either the cytolytic function of CD8+ T cells or natural killer T-cells or a defect in innate immunity. Secondary HLH can be caused by infection, including Epstein-Barr virus (EBV), malignancy, or autoimmune disorders [2-4].

HLH can be diagnosed when one of two criteria is fulfilled: a molecular diagnosis consistent with HLH or five out of eight diagnostic criteria are met. The diagnostic criteria include the following: fever, splenomegaly, cytopenia, hypertriglyceridemia and/or hypofibrinogenemia, hemophagocytosis in bone marrow/spleen/lymph nodes, negligible natural killer cell activity, elevated ferritin ≥500 μg/L, or soluble CD25 ≥2400 U/mL [1,3,5]. In addition, there is an online diagnostic tool referred to as the HScore, which shows the probability of HLH that may aid in diagnosis. Parameters of the HScore include underlying immunosuppression, temperature, organomegaly, cytopenia, ferritin level, triglyceride level, fibrinogen level, aspartate aminotransferase level, and hemophagocytosis on bone marrow aspirate [6].

The current treatment of HLH is the HLH-2004 protocol over a span of 40 weeks. This protocol was based on the HLH-94 protocol and includes dexamethasone, etoposide, cyclosporine A, and intrathecal methotrexate and prednisolone [6,7].

Although EBV is a frequently transmitted virus worldwide and has minimal effects on immunocompetent individuals, it poses a serious risk to patients with genetic diseases that predispose them to HLH. We report a rare case of HLH secondary to EBV diagnosed in a 27-year-old male, who was treated with the HLH-2004 protocol.

## Case presentation

A 27-year-old Hispanic male with no significant medical history presented to the emergency department with a chief complaint of generalized fatigue persisting for two weeks. The patient also reported the presence of jaundice for one month. He had previously sought medical attention at the clinic and was discharged with prescriptions for ciprofloxacin and metronidazole due to concerns of liver infection. Additionally, the patient reported symptoms of myalgia, nausea, vomiting, right upper quadrant abdominal pain, and dizziness.

On initial physical examination, the patient showed hypotension, tachycardia, tachypnea, and fever. Laboratory investigations revealed significantly elevated total bilirubin, direct bilirubin, aspartate aminotransferase (AST), and alanine transaminase (ALT) (Table 1). 

**Table 1:** Laboratory values obtained at initial hospital admission ALT: alanine transaminase; AST: aspartate aminotransferase; BUN: blood urea nitrogen; EBV: Epstein-Barr virus; Hgb: hemoglobin; Hct: hematocrit; PLT: platelets; PT: prothrombin time

Lab value	Day 1	Day 2	Day 3	Reference value
WBC	2.9	2.4	2.9	3.2-9.7 10E3/uL
RBC	2.84	2.87	3.3	4.08-5.70 10E6/uL
Hgb	7.2	8.2	9.2	13.1-16.8 g/dL
Hct	20.8	25	25.7	38.2-48.4%
PLT	55	29	32	130-351 10E3/uL
Neutrophils%	77	-	-	41-75%
Lymphocytes%	13.8	-	-	16-46%
PT	24.6	43.7	15.3	11.7-14.3 seconds
INR	2.2	4.5	1.2	0.9-1.1
Fibrinogen	112	-	185	204-466 mg/dL
BUN	21	23	34	7-20 mg/dL
Cr	0.7	0.69	0.47	0.66-1.25 mg/dL
AST	138	-	-	0-50 U/L
ALT	243	-	-	0-50 U/L
Alkaline Phosphatase	307	-	-	38-126 U/L
Triglycerides	257	-	-	<150
Total bilirubin	26.8	19.3	25.3	0.2-1.3 mg/dL
Direct bilirubin	14.1	-	23.1	0-0.4 mg/dL
Total protein	6.2	4.6	4.9	6.3-8.2 g/dL
Ferritin	>10,000	-	-	17.9-464 ng/mL
EBV DNA PCR	56300	-	-	0-500 IU/mL
sCD25	-	-	Elevated	0.45-3.83 ng/mL

**Figure 1:**
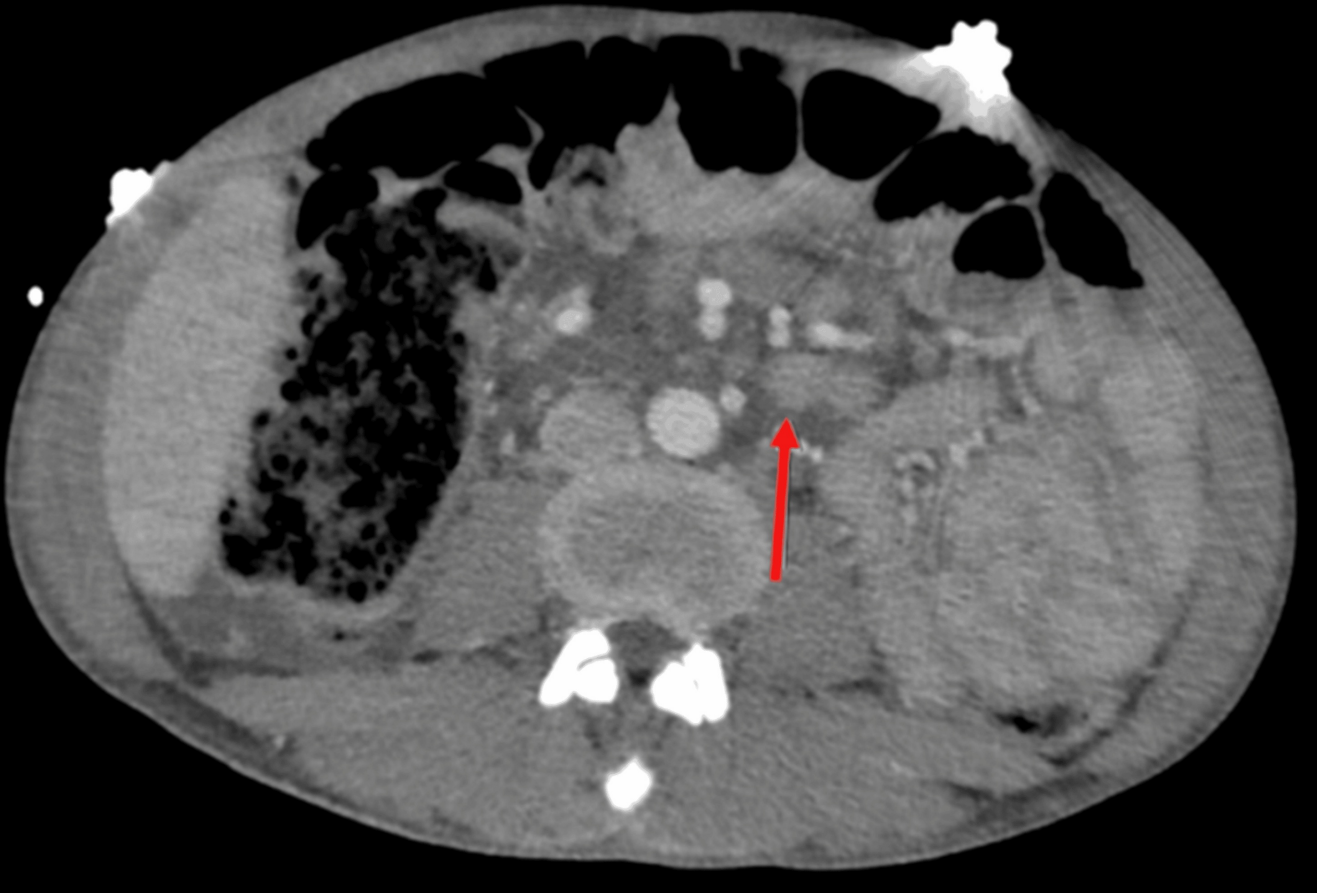
Contrast-enhanced computed tomography scan of the abdomen in the axial plane, showing mesenteric and retroperitoneal lymphadenopathy

Computed tomography (CT) with contrast of the abdomen and pelvis, along with gallbladder ultrasound, was performed. The CT revealed hepatomegaly with a length of 23 cm, accompanied by periportal edema (Figure 1). Pericholecystic fluid, measuring up to 8mm, was observed without indication of common bile duct obstruction. Additionally, there was persistent free fluid in the abdomen and pelvis, splenomegaly measuring 16.4 cm, and mild-to-moderate mesenteric lymphadenopathy. The ultrasound displayed gallbladder wall thickening with mild pericholecystic fluid, raising concern for possible cholecystitis.

During the consultation, the gastroenterology (GI) and general surgery departments were involved. The GI specialist requested an EBV polymerase chain reaction (EBV PCR), cytomegalovirus (CMV) PCR, and hepatitis panel and advised against performing an endoscopic retrograde cholangiopancreatography (ERCP). Additionally, vancomycin was initiated. The general surgery department confirmed that surgical intervention was not necessary at the present time. They suggested that, if cholecystectomy were to become necessary, the patient should be transferred to hepato-pancreato-biliary (HPB) specialists.

A consultation was initiated for HPB specialists, who observed abnormalities in the patient's liver. An interventional radiology consultation was requested for the placement of a cholecystostomy tube, and a consultation from hepatology was also sought. Additionally, a hepatitis profile and screening for drug, alcohol, and toxic substances were requested.

Upon evaluation, the hepatology specialist established a preliminary diagnosis of acute hepatitis due to CMV infection. However, the CMV viral load was insufficient to warrant initiation of treatment. In the interim, the patient was commenced on midodrine and administered albumin, pending the results of the hepatitis workup and liver biopsy.

The patient underwent interventional radiology for a liver biopsy, revealing a mixed inflammatory infiltrate with non-monoclonal large plasma cells. Subsequently, the patient was diagnosed with interface/autoimmune hepatitis and initiated on prednisone. At this stage, an EBV stain was advised.

At the time of discharge, the patient received a prescription for prednisone and was advised to schedule an appointment with the hepatology department the following week. Subsequently, the patient was readmitted to the hospital one week after discharge when routine laboratory tests revealed a low hemoglobin level of 6.6, necessitating a blood transfusion. A new CT scan was requested, and the hepatology specialist recommended a consultation with the hematology/oncology specialist. The hepatology specialist raised concerns about the potential for bone marrow suppression if antiviral medications were administered.

The hematology/oncology department advised to maintain the patient's steroid treatment, commence antiviral medication, and conduct an iron workup along with a bone marrow biopsy. Initially, the bone marrow biopsy yielded negative results for malignancy; however, upon reassessment, it revealed hemophagocytosis. The EBV PCR test returned an initial viral load of 23,900.

Suspicion of hemophagocytic lymphohistiocytosis prompted further evaluation, necessitating tests for ferritin, LDH, and triglycerides. The patient exhibited six out of eight HLH-2004 diagnostic criteria, including splenomegaly, fever, cytopenia in three lineages, hypofibrinogenemia, hyperferritinemia, and positive sCD25. Additionally, they showed an HScore (HLH-probability calculator) of 233, giving a 98-99% probability of hemophagocytic syndrome. Consequently, the patient was diagnosed with HLH and underwent assessments for malignancy through PET scan, flow cytometry, and monoclonal spike evaluation.

A PET scan yielded non-diagnostic results, and no specific target for biopsy was identified. Subsequently, the patient underwent a robotic mesenteric lymph node biopsy to exclude the presence of lymphoma. The biopsy results revealed no evidence of malignancy. The patient received a bone marrow aspiration, biopsy, and flow cytometric evaluation revealing severe anemia, mild relative and absolute lymphopenia, minimal thrombocytopenia, and phagocytosis of both red cells and lymphocytes by histiocytes, concluding that the underlying significant disease process in the patient was HLH, causing clinical symptoms and hematologic abnormalities (Table 2). The HLH-2004 treatment protocol was started, and the patient initially responded; however, after the third day, he started to deteriorate medically and clinically, resulting in the patient's demise. 

**Table 2:** Bone marrow aspiration values obtained at subsequent hospital admission

Value	Results	Reference value
White Blood Cells	5.9	3.1-9.7 THOU/mm4
Red Blood Cells	1,47	4.08-5.70 mil/mm3
Hemoglobin	4.5	13.1-16.8 g/dL
Hematocrit	12.2	38.2-48.4%
Mean Corpuscular Volume	83.3	80.1-98.5 fL
Mean Corpuscular Hemoglobin	30.7	27.1-34.2 pg
Mean Corpuscular Hemoglobin Concentration	36.9	31.7-35.2 g/dL
Red Cell Distribution Width	17.9	12.3-16.3%
Segmented Neutrophils	76.8	40.6-75.3%
Lymphocytes	15.8	16.1-45.7%
Monocytes	5.4	3.7-12.2%
Eosinophils	0.1	0-6.3%
Basophils	1.9	0.1-1.3%
Platelets	128	130-351 THOU/mm^3^
Polymorphonuclear Lymphocytes	4	6-12%
Band Neutrophils	9.2	9.5-15.3%
Metamyelocytes	3.2	9.6-24.6%
Myelocytes	7.2	8.2-15.7%
Promyelocytes	0.4	2.1-4.1%
Myeloblasts	0.8	0.2-1.5%
Rubriblasts	1.2	0.2-1.3%
Prorubricytes	6.4	0.5-2.4%
Rubricytes	38	13.1-30.1%
Metarubrucytes	3.2	0.3-3.7%
Plasma Cells	12.4	0.4-3.9%
Myeloid to Erythroid Ratio	0.6	1.5-3.3%
Megakaryocytes	Present	-

## Discussion

HLH is an uncommon and highly fatal hyperinflammatory clinical syndrome. It is characterized by an uncontrolled inflammatory response and excessive immune cell activation where abnormal macrophages and T-cells release many cytokines, leading to tissue damage and multiple organ failure [5,7]. This disease can be classified into two categories: primary, or genetic, lymphohistiocytosis, and secondary lymphohistiocytosis.

Primary lymphohistiocytosis is more prevalent in children and young adults, with an estimated annual incidence of 1.2 per million children. Secondary lymphocytosis is more common in adults due to infections such as EBV, malignancies such as T-cell lymphomas, and rheumatic disease; the prevalence is not well established [7,8]. Even though EBV is a frequently transmitted virus worldwide with minimal effects on immunocompetent individuals, EBV poses a serious risk to patients with genetic diseases that predispose them to HLH [9]. Other viruses such as CMV, dengue, and COVID-19 have also been reported [10]. Secondary HLH mostly follows EBV infection, but may also be induced by malignancies known as malignancy-associated HLH or M-HLH. Non-Hodgkin lymphomas (NHL) are a risk factor for HLH, with a reported prevalence of HLH up to 20% in some subtypes [11].

HLH is a clinical syndrome that usually presents with vague signs and symptoms, which makes the diagnosis challenging. Patients typically present with fever, hepatosplenomegaly, cytopenias, hepatitis, coagulopathy, central nervous system disturbances, and rare complications [11]. The diagnosis in adults is based on the HLH-2004 diagnostic criteria: a molecular diagnosis consistent with HLH or meeting five or more of eight diagnostic criteria such as fever, splenomegaly, cytopenias affecting two or more of three lineages in peripheral blood, hypertriglyceridemia and/or hypofibrinogenemia, hemophagocytosis in bone marrow, spleen or lymph nodes without evidence of malignancy, low or no NK cell activity, hyperferritinemia, and sCD25 >2,400 U/mL [11]. Because of the variety of symptoms, the differential diagnosis between a severe infection and HLH can be challenging.

There is a rapid clinical deterioration in treatment-naive EBV-infected patients; therefore, it is crucial to make a prompt diagnosis and initiate treatment. According to the HLH-2004 guideline, the recommended regimen includes immunosuppressive agents such as cyclosporine, etoposide, and dexamethasone, as well as adjunctive treatment with rituximab and splenectomy [12]. In addition, the administration of prophylactic treatment for fungal infections and *Pneumocystis jirovecii *should be given to high-risk patients with depleted leukocyte function due to the HLH treatment protocol. Bone marrow transplantation may be necessary for patients with refractory EBV-HLH, but the ideal timing of transplantation is often unclear. Mortality is high (95%) if left untreated. Prognosis in adults is generally worse than in pediatric patients, along with its diagnostic challenge due to its various clinical presentations [13].

## Conclusions

Our case report describes a case of HLH in a young adult presenting with vague symptoms and positive EBV serology. Due to its sparse incidence in adults, HLH can be easily misdiagnosed, leading to delayed and inappropriate treatment. We emphasize the significance of prompt diagnosis and treatment with immunosuppressive therapies and rituximab due to high mortality, especially in adults. By increasing awareness of HLH, a more rapid diagnostic workup and new therapeutic approaches can improve patients’ prognosis. Broadening the array of differential diagnoses keeps the physician more open to diseases that less frequently occur, leading to more accurate and successful treatment.
